# Supplementary material for: Explosion of formulaic research articles, including inappropriate study designs and false discoveries, based on the NHANES US national health database
Source: PLoS Biol. 2025 May 8;23(5):e3003152. doi: 10.1371/journal.pbio.3003152 (PMC12061153; doi:10.1371/journal.pbio.3003152)
Supplement: Attachment — Submitted filename: ReviewResponse_PBIOLOGY-D-25-00160R1_5Mar2025.pdf [file pbio.3003152.s004.pdf]

School of Health Sciences
University of Surrey
Guildford
Surrey
GU2 7XH, UK

3 March 2025

Dear Editorial Team

Reviewer Response: PBIOLOGY-D-25-00160R1

Many thanks to the reviewers for their constructive feedback. It is our pleasure to provide a point-by-point response below. Our replies are indented in blue text.

Reviewer #1: [identifies herself as Jennifer A. Byrne]

This manuscript describes an important problem, namely the possible exploitation of NHANES-based research data for the scaled production of low value research manuscripts. The manuscript describes a steep recent rise in the number of articles published using NHANES data. It is important for these results to be communicated rapidly, as it would seem that the numbers of such manuscripts could continue to rise, possibly overwhelming editorial and peer review processes at some journals.

I should specify that my expertise lies outside the types of statistical analyses performed, so I wasn't able to critically evaluate all the data.

Major issues:

Page 6: I strongly suggest to not cite the studies listed in Tables 2 and 3, but to instead use PubMed ID's within Tables and text. Paper mills are likely to value to citations to their work, so citing many problematic papers inadvertently supports this model.

> These studies are now referred to by PMID alone (references have been removed from the tables and the text), also in the Discussion. As long as this meets Editorial requirements for the journal, we agree that this is a much better solution.

Page 10: "The challenges analysed here are different to those for manufactured manuscripts using falsified data"- this warrants further discussion. Arguably, some challenges are the same, i.e. fabricated and genuine yet low value/derivative manuscripts can both report unreliable results, both classes of manuscripts waste editor and peer reviewer time. It would be worth explaining the similarities and differences between manuscripts generated from AI-ready datasets versus those that are entirely manufactured from fabricated data, so that any specific issues are clearer.

We have added a small amount of additional text to cover this point. The main issue in our eyes is that using 'genuine' data adds a veneer of respectability and authenticity to these manufactured manuscripts which they do not warrant. When combined with apparently appropriate statistical methods, in isolation such papers may seem reasonable. The added text is also reproduced below.

"Whilst there are challenges in common with those posed by manufactured manuscripts using falsified data, data dredging from open access datasets creates different issues (as the statistics and data may – in isolation – be sound), and will require different countervailing measures."

Page 10: "dedicated statistical reviewers"- many journals would indeed benefit from statistics reviewers, however, their time could be easily consumed by low value manuscripts. The authors then call for better manuscript screening processes, which are essential for expert reviewers to focus attention on quality submissions. This point should be expanded, e.g. by referencing recent publications.

Paul M Stewart, "Houston We Have a Problem….": Raising the Quality and Authenticity of Manuscripts Submitted to JCEM, The Journal of Clinical Endocrinology & Metabolism, 2025;, dgaf036, https://doi.org/10.1210/clinem/dgaf036

(this very recent editorial mentions similar manuscripts to those described here), also

Stender, S., Gellert-Kristensen, H. & Smith, G.D. Reclaiming mendelian randomization from the deluge of papers and misleading findings. Lipids Health Dis 23, 286 (2024). https://doi.org/10.1186/s12944-024-02284-w

More emphasis should be placed on the importance of desk rejections to save editorial and peer reviewer time, as per Stender et al. (2024). Stender and colleagues have proposed a peer reviewer template to recommend rejection of low value 2SMR manuscripts- this could be built upon to provide similar resources for reviewers of NHANES-based manuscripts. This would seem to be a valuable addition.

Thank you, both of these references have been added, and we appreciate the addition of these up-to-date and helpful resources. We have also added a 'template' reply under Suggested Action 3 in the Discussion, and have specified that this will be of particular use for desk rejections (this issue has also been raised by other reviewers).

"3. Publishers should emphasise the importance of desk rejection to reduce the burden on peer reviewers. Stender et al. (2024) suggested the use of a template for efficient rejection of papers using formulaic approaches to two sample mendelian randomization, which we have adapted here as an example"

Minor issues:

Page 1: Manuscript subtitle "Unethical research practices"- could this be "Questionable", "Problematic"? Research ethics doesn't seem to be a focus of the manuscript.

The reviewer is correct that research ethics can have a specific meaning, so we have changed it to 'problematic', but in a more general sense of the word, these research practices are definitely unethical in our view!

Page 2: The abstract is quite long and includes some details that could be omitted.

The Abstract length is within journal guidelines, and we are all too aware that it will often be the only part of the manuscript that is read, so we have made small reductions only. If there are specific details that the reviewer thinks should be removed, we would be happy to do so.

Figure 1: The authors could check the n values shown, eg 426-6 does not equal 417.

We have corrected the PRISMA chart, thank you for spotting the error!

Figure 1: legend refers to a "systematic review", whereas the text refers to "meta-analysis" (lines 124, 221, 307).

Changed to refer to "*four-phase flow diagram of the article identification process*".

Figure 2: The legend does not describe the colours used, some of which are used more than once. Are the colours significant?

The colours are used for visual clarity only and the legend now states this, "*Colours are used to assist visual separation of blocks and do not represent data types or categories*".

Page 5: "chow test" (used twice), "Chi-square test" are not mentioned in the Methods.

Reviewer two requested the removal of these calculations, on the basis that the trends speak for themselves and it was not clear that a prior hypothesis was being tested (rather than statistics being selected *post-hoc* to buttress an argument). We have removed these tests and hope that the other reviewers will still find the numbers persuasive without the p-values!

Page 5: "biobank" is used as a keyword to show recent increases in data-driven research (Figure 3B), yet most human health biobanks provide biospecimens and associated data, and hence support laboratory research that's not primarily data-driven. The authors could therefore rethink the use of "biobank" as a control keyword.

The reviewer makes a reasonable point, it was not easy to identify a control keyword that would exclude the manufactured manuscripts mentioned here or in Stender et al. (2024). We tried 'biomarker' and 'health data' and all showed much the same trends. On balance, we agree that 'biobank' is not perfect but think it is adequate to make the point.

Figure 3: Colours used are not defined.

We have removed the colours as previously they corresponded to the chow-tests.

Figure 5: Suggest "Publication count" for Y axis, also "publications" in legend.

Added.

Page 8: Suggest inserting a paragraph break at line 194- paragraph is currently quite long.

Added at "In terms of productivity" as requested.

Page 10: "often peer reviewers will be the last"- this is an important point, however the cited reference from 2008 would have been unlikely to specifically refer to paper mills. This sentence could be reworded for clarity.

The reference has been moved to make clear it is a citation relevant to the importance of peer review generally, rather than paper mills specifically.

Page 10: "early warning lists"- it's unclear what's intended here. Some readers will associate this term with the CAS journal early warning lists.

> We have removed this sentence, as we agree it is likely confusing. Originally we had wanted to suggest early warning lists of problematic authors, but on reflection this seems unlikely to be practicable. It is far more likely that success will be had by targeting the templates (which are easy to identify and relatively few in number) than authors (which are very numerous).

Reviewer #2: [identifies himself as Nikolaus C Netzer MD PhD]

I completely support this manuscript by all means, agree in all points with the authors and think it is extremely important to get the message out. I have a few minor requests based on my own experience with the topic.

1. I know that GB is not a part of the EU anymore, still research strings are tight. I would shortly discuss in the paper the EU AI in research act. Especially since some influential economical experts recently criticize the EU for being to restrictive with AI regulations and that it would therefore harm the economical and scientific progress and success of EU countries. I think this critizism is only money driven and lacks any ethical aspects of the topic (Netzer NC. Artificial intelligence - the Janus-faced tool in our hands. Sleep Breath. 2024 Oct;28(5):1861-1862. doi: 10.1007/s11325-024-03129-7. Epub 2024 Aug 5. PMID: 39098968; PMCID: PMC11449974; https//research and innovationec.europa-eu./research area/industrial research and innovation/ Artificial Intelligence (AI) in Science - European Commission (europa.eu)).

> Thank you, this reference has been added. We agree on the AI point, there are also some wider issues that are less black / white with the FAIR Guiding Principles. Whilst probably out of scope for this paper, at some point policymakers will want to consider whether the trade-offs of open access are worth it (we are inclined to the view that access will need to be made conditional in the long run, which is less than ideal, but the lesser of two evils). We have also added a reference to the EU Commission's work in the area, but not added discussion of their specific initiatives, as this felt a little too much like drift from our focus.

2. The problem with single parameter correlations is not only that they might deliver false results. I found a MR NHANES manuscript as reviewer, where the AI hallucinated by simply turning the hypothesis around from two single parameters. The first result was NHANES data of depression in subjects correlated with data of sleep apnea and the conclusion of the paper (the AI) was sleep apnea leads to depression. That's scientifically not proven (most patients have daytime sleepiness, but do not develop a depression from that) but possible. Then I got the same manuscript again to review with the authors in reversed order, turning the conclusion around (same correlation), that depression leads to sleep apnea. Physiologically and pathophysiologically total nonsense. I described that problem in the above listed editorial in Sleep and Breathing.

Maybe you want to build that problem with reversed single parameter correlation into your arguments respectively discussion or introduction.

> This is an interesting point and inevitable if the 'factory' is deriving every possible pairwise association, and the below text has been added.
>
> *"Some variables were included as both predictors and as outcomes, for example C-reactive protein (CRP) was investigated as being associated with periodontitis (PMID*

37481511), but in another study a health behaviour index was investigated as a predictor of elevated CRP levels (PMID 38628050). In the most extreme case additional manuscripts could be generated by simply reversing dependent and independent variables in the statistical analysis, increasing the number of combinations of predictors and outcomes with no physiological justification or hypothesis."

Reviewer #3: [identifies herself as Dorothy Bishop]

Summary

After a move to encourage researchers to adopt open data, it is becoming clear that unintended negative consequence is data dredging of open datasets that leads to pointless analyses being published. The authors demonstrate this phenomenon with the NHANES dataset, showing that there has been a rapid growth in papers reporting associations between single predictors and single outcomes. A high proportion of the recent paper originate from China. The authors argue that this research is flawed because it does not take into account the multifactorial origins of most health conditions, and that AI-ready datasets may be misused by paper mills. They conclude with some recommendations for overcoming this situation.

Overall evaluation

The authors have conducted a systematic analysis of papers from the NHANES dataset, and provide useful empirical evidence to support the view that single-factor analyses have skyrocketed in recent years. This is useful but I see two aspects that I am not convinced by and that I'd like to see addressed: (a) the need for multifactor analyses and (b) the involvement of paper mills. In addition, I think the recommendations could be a lot stronger.

Our responses are below, but in general, (a) we agree that multifactorial analyses are not complete solutions, but best practice is surely to take a broader approach, and (b) we have been careful in our choice of language on paper mills, but think there is sufficient evidence here to at least highlight their potential involvement. We have also strengthened the recommendations in line with the reviewer's request.

Multifactor analysis

While I agree that single factor accounts of most health conditions are implausible, I'm not sure the problem identified here would be solved by researchers adopting multi-factorial analyses. It could be argued that it would just make the problem worse, given that the number of potential associations increases multiplicatively as more factors are included in the analysis. In other words, unless analyses are pre-registered and theoretically or empirically motivated, encouraging authors to just include yet more variables in the analysis will make matters worse rather than better, because the chance of false positive findings increases dramatically. So I think the focus on single vs multiple factors is a bit misleading here.

The reviewer makes a good point, and indeed it was not our intention to say that multifactorial analysis would *solve* the problem. Our current wording is that *"Best practice is to conduct research in a comprehensive manner, or run the risk of research not being translational, [20] with real costs to healthcare systems and patients. [21,22]"* We think that this is a defensible point of view; our target is to highlight the issue with single-factor analyses which conflate general indicators (e.g. inflammation) with diseases and imply specificity which does not exist.

Involvement of paper mills

Paper mills were mentioned at several points during the article, but no evidence was provided for their involvement in the explosion of single factor papers. Given that these mostly originate in China, where the incentives for publishing in Western journals have changed dramatically, it's not clear that we are necessarily seeing the result of paper mill activities. See this article that discusses how incentives can lead to misconduct but which does not feature paper mills as a mechanism:

Zhang, X., & Wang, P. (2025). Research misconduct in China: towards an institutional analysis. Research Ethics, 21(1), 76-96. https://doi.org/10.1177/17470161241247720

Diagnosing paper mills is not a straightforward business, and I can see it would be beyond the scope of this article to attempt to do such an analysis. Nevertheless, it might be feasible to look at two indicators: the geographical diversity of authors, and the use of non-institutional emails, both of which have been identified as red flags for paper mills:

Van Noorden, R. (2023). How big is science's fake-paper problem? Nature, 623(7987), 466-467.

Alternatively, the paper could be rewritten to make it clear that the statements about paper mills are conjecture and not supported by evidence.

> We have added the two references, and agree that these are helpful context. We have also added some text on incentives in China to Discussion. On the language, we have taken particular care, and believe that at no point have we used unwarranted conjecture. For example, we frequently talk about "*an approach that can easily be adopted by paper mills*", which is not conjecture, it is an approach that *can indeed* be adopted. We also refer to "*risks of data exploitation by paper mills*", again not conjecture, there *are* risks. Similarly, we say that "*It is also plausible that the use of high-quality legitimate datasets such as NHANES is part of a strategy that may assist paper mills in avoiding detection*" – we do not consider it conjecture to flag that this is a *plausible* approach that *may* be employed by paper mills. We have added some additional language, for example "*Second, our analysis is not intended to criticise individual manuscripts or attribute them to paper mills*" and hope that this is enough to meet the reviewer's concerns. Of course, if there is a specific statement about paper mills that the reviewer feels is too conjectural / problematic then we would be pleased to amend it.

Recommendations for action

An additional recommendation would be to provide access to just half of the dataset, which could be used for exploratory analysis. The researcher would then have to show that the results replicated in the other half - a kind of cross-validation sample.

Also, the OpenSafely model used for accessing NHS data provides good protection against data dredging: Nab L, Schaffer AL, Hulme W, et al.. OpenSAFELY: A platform for analysing electronic health records designed for reproducible research. Pharmacoepidemiol Drug Saf. 2024; 33(6):e5815. doi:10.1002/pds.5815

Requiring authors to pre-register their research hypothesis and making data access contingent on this sounds similar to the option 2 discussed by the authors, but I was not clear how far that required explicit specification of a hypothesis.

In genetics, where data dredging led to a decade or so of non-replicable candidate gene studies, it became mandatory to replicate the results in a fresh sample. This is similar to the cross-validation idea above, but may be more suited to a case where the sample size is small.

Thank you, we agree that there is substantial merit in these ideas, and have added the following text to suggestion two in the Discussion "*in-line with similar approaches such as pre-registration of studies or models such as OpenSafely employed by the UK's National Health Service.*" We have also added the suggested references.

We have not gone too far into additional discussion of policymaker responses and the landscape of government and supranational agencies, beyond some signposting references (e.g. to the EU Commission, as suggested by another reviewer). It is also hard to know when to stop, because arguably these trends challenge the FAIR Guiding Principles and the concept of Open Data, a whole new topic. We did not want to drift from the paper's goal, which is to highlight the specific problem of mass-manufactured manuscripts targeting open data sources, using NHANES as an example, so we have tended to focus our actions on things that can be done relatively quickly by practitioners. But we agree that a wider-ranging policy paper would generate substantial interest and have added some text to this effect at the end of Discussion.

The kind of rapid growth in a methodology described here is reminiscent of what has been reported in other areas: Stender, S., Gellert-Kristensen, H., & Smith, G. D. (2024). Reclaiming mendelian randomization from the deluge of papers and misleading findings. Lipids in Health and Disease, 23(1), 286. Those authors had pretty stringent recommendations for journal editors that might be applicable also in this case.

We have included a number of additional references to Stender et al. and also adapted the suggested template / other recommendations. We agree that the work of Stender et al. is excellent and effectively see two 'formulaic' templates, one for 2SMR and one for NHANES. Likely, any large scale dataset with an API will be targeted in the same way and new templates will emerge.

More minor points

p 2 line 26, "Second,..." this is not a sentence; just needs rewriting

Rewritten, thank you for picking up on this.

line 108. I wasn't sure what was meant by the sentence starting "There was a substantial.."

We agree that this was clumsily and confusingly written, and have changed the sentence to start "*In terms of trends over time, an... average of...*"

[On the] paragraph using Chow test and chi square test on p 5. The authors have criticised others for reporting p-values after data dredging, but the statistical results reported here have a similar flavour. That is, it is not clear that there was a hypothesis, in which case p-values seem inappropriate. I think the trends are very obvious in any case and that purely descriptive data would be fine here.

Our hypothesis was a simple one, that the change in trend was statistically significant and not just 'noise' in a time series, and the Chow test was used to test this. But we also agree that there is a 'hint' of post-hoc digging for statistically significant results to buttress

our prior beliefs. We have removed the Chow test and Chi-squared tests accordingly and hope that readers will be persuaded by the data without them.

p 6, line 146. I was puzzled as to how the FDR correction was applied. This eventually became clear after reading the methods. It would make more sense if the Methods preceded the Results, if the journal allows this. In any case, it seems that the use of FDR correction involves some strong assumptions that may not be justified. One issue is that we don't know how many tests were conducted by went unreported. And as the authors note later on, determining the number of potential hypotheses is not trivial. This is one reason why I think the key issue is not so much whether you have single factor or multi factor analyses, but rather whether you have clearly defined a priori hypotheses.

The journal format is for Methods after Results. We have made some slight modifications to the text to improve the flow [Line 146].

"The individual studies did not employ false discovery correction, but taken together, represent multiple hypotheses. To compensate for this…"

p 8 line 172. Again, I don't like referring to a "statistically significant increase" when no hypothesis has been stated. Sorry for being pedantic, but I'd really prefer to just have a descriptive statement here, maybe referring to a N-fold increase.

No need for apologies, better to get it right. We have removed the post-hoc statistical tests and hope that the data speak for themselves.

p 8 line 181. para on single-factor design. This is where I think you need to be careful to not encourage multifactor data dredging. A multifactor analysis should be motivated either by reference to prior literature and/or by theoretical considerations; just throwing lots of variables into the analysis would make matters worse I think.

Whilst we understand the point the reviewer is making, we think that taking the paper as a whole, it does not encourage multifactor data dredging. We have made some additional small text modifications to make this clear.

p 10 ;I suggest new header at end of para 1.

We have added a new heading, "Recommendations", thank you for the suggestion.

Reviewer #4:: [identifies himself as Stefan Stender]

This study analyzed the rapid growth in publications stemming from single-factor association analyses of the publicly available National Health and Nutrition Examination Survey (NHANES) cohort. A literature search using a set of criteria identified 341 NHANES-derived papers from 2014-2024, each proposing an association between a predictor and a health condition. The number of papers per year is rising rapidly in recent years. The authors find evidence of data dredging and hypothesizing after results are known ('HARKI'ing'). They highlight the potential of these formulaic NHANES-papers being made (wholly or partly) by AI-methods and/or paper-mills.

Finally, the authors describe a set of best practices to address the concern of this type paper flooding the literature. The study deals with an important and very timely topic. The paper is well written. The findings and arguments are compelling.

I have a few minor comments:

1) The search criteria are quite specific: 'NHANES AND (correlation* OR association*) AND (cross-sectional OR population)'

I realize this is probably required to limit the number of hits. However, I think it's worth reporting the total number of NHANES papers identified just with simple search 'NHANES' by year:

This is striking. The number per year was rising steadily from 2000-2017, plateauing from 2018 to 2023 at around 4700 per year, and then spiking to N=7818 in 2024!

After 60 days of 2025 we are already at N=1485. So, the projected number of NHANES papers for 2025 is probably > 10000! In other words, there is evidence that the explosion in NHANES papers is on a much larger scale than the numbers reported from the articles identified by the authors using the narrow search criteria. I think this is worth pointing out.

We have added this commentary. The reviewer is quite correct that we wanted to dig into some specific issues, like what would happen if we checked whether the authors had used all data, or selective data, and this was only achievable by targeted study. The following text has been added.

"The emphasis here is to identify the problem and make recommendations for stakeholders, rather than to provide a definitive list of potentially problematic works, but it should be noted that the total number of NHANES manuscripts identified by a simple search of the PubMed database (searching for NHANES at https://pubmed.ncbi.nlm.nih.gov/, accessed 3 March 2025) increased from 4926 in 2023 to 7876 in 2024. This is suggestive that the scale of the problem could reach thousands of manuscripts, rather than hundreds. Such estimates would be concordant with the trend previously identified in the field of Mendelian randomization, another area which has seen a tsunami of formulaic manuscripts."

2) The issue with NHANES papers was to my knowledge first noted in an editorial in 2024:

Netzer, N.C. Artificial intelligence – the Janus-faced tool in our hands. Sleep Breath 28, 1861–1862 (2024). https://doi.org/10.1007/s11325-024-03129-7

This editorial should be cited.

We have added this reference, thank you for the addition.

3) A very similar issue is plaguing the field of Mendelian randomization (MR), where we see an explosion in number of papers using publicly available summary data in recent years. This explosion is driven by papers based on two-samples MR (2SMR), where both the exposure and outcome data are based on summary statistics from published GWAS. The rise is entirely driven by papers from China, and there is evidence that some are being produced by paper-mill like factories and using AI. The mass-produced 2SMR has been the focus of several recent editorials and comments: PMID: 39244551, PMID: 39311417, PMID: 39407214. It would be relevant to highlight this analogous situation in the discussion of the present manuscript.

We have added these references, and also included some adapted recommendations, including a template for Desk Editor rejection. In fact, the reviewer will note that his work is highly recommended by the other reviewers, and we have incorporated a number of valuable references to it.

With thanks again to all reviewers for the comprehensive comments, and the generally positive sentiment. We hope that our work can make a contribution to this important issue.

Best wishes,

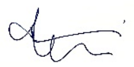

Matt Spick
Lecturer in Health and Biomedical Data Analytics
School of Health Sciences
