# Supplementary material for: Explosion of formulaic research articles, including inappropriate study designs and false discoveries, based on the NHANES US national health database
Source: PLoS Biol. 2025 May 8;23(5):e3003152. doi: 10.1371/journal.pbio.3003152 (PMC12061153; doi:10.1371/journal.pbio.3003152)
Supplement: Attachment — Submitted filename: ReviewResponse_PBIOLOGY-D-25-00160R2_22Mar2025.pdf [file pbio.3003152.s005.pdf]

School of Health Sciences
University of Surrey
Guildford
Surrey
GU2 7XH, UK

22 March 2025

Dear Editorial Team

Reviewer Response: PBIOLOGY-D-25-00160R2

Many thanks to the Editorial Team for their constructive feedback. For completeness, I have detailed our responses to each of the points below. I also thought I would mention here that the University of Surrey has an overall agreement with PLOS Biology for Open Access fees. If this is not your understanding, not a problem, we can cover them. But I thought it might be helpful to mention it now so that if necessary you can let us know if it does / does not apply here.

Editorial Comments

Please change your Title to something that brings the importance of the advance more to the fore. We suggest: "Explosion of formulaic research articles, including inappropriate study designs and false discoveries, based on the NHANES US national health database".

> We have changed the title as requested.

Please address my Data Policy requests below; specifically, we need you to supply the numerical values underlying Figs 3AB, 4, 5, either as a supplementary data file or as a permanent DOI'd deposition.

Please cite the location of the data clearly in all relevant main and supplementary Figure legends, e.g. "The data underlying this Figure can be found in S1 Data" or "The data underlying this Figure can be found in [link to repository]

> For Figures 3A and 3B, we have added a new Table S4 to Supplementary Material and the legend for Figure now refers to this. For Figure 4, the data are taken from the pivot tables in Supplementary Material, Tables S2 and S3, and the legend of Figure 4 now states this as the source explicitly. Similarly, for Figure 5, the data come from Table S1 and the legend now states this as the source, and we have added some additional text to the legend to make the source of the data more clear.

Please make any custom code available, either as a supplementary file or as part of your data deposition.

> No customised code was used and the Data Availability Statement states that "All code libraries were used without modification or customization".

Your current Financial Disclosure states, “ Charlie Harrison and Reyer Zwiggelaar were supported by the Biotechnology and Biological Sciences Research Council (BB/Y006933/1). The funders had no role in study design, data collection and analysis, decision to publish, or preparation of the manuscript.” Please indicate by return email the full and correct funding information for your study and confirm the order in which funding contributions should appear. Please be sure to indicate whether the funders played any role in the study design, data collection and analysis, decision to publish, or preparation of the manuscript.

> I have also responded by return email, and the disclosure “Charlie Harrison and Reyer Zwiggelaar were supported by the Biotechnology and Biological Sciences Research Council (BB/Y006933/1). The funders had no role in study design, data collection and analysis, decision to publish, or preparation of the manuscript.” is correct. I have checked that it says this in the manuscript and in the Editorial System, if I have missed anywhere, it should read as above and I apologize.

Please refer to the following article for PLOS Biology's best-practice recommendations for data presentation.

> This is very useful and I agree with the recommendations wholeheartedly, but I think all our data are compliant.

With thanks again to the Editorial Team. The process for this submission has felt very painless from our end, and we very much appreciate your efforts and those of the reviewers. Especially, we appreciated that four expert-in-the-field reviewers were recruited, it makes a difference to have the work engaged with by people that have genuine expertise and enthusiasm for the topic. It isn’t always the case!

Best wishes,

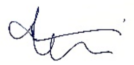

Matt Spick
Lecturer in Health and Biomedical Data Analytics
School of Health Sciences
